# Telemedicine applications in general medicine - a structured review of the evidence

**DOI:** 10.3389/fmed.2026.1771532

**Published:** 2026-04-24

**Authors:** Philip Marahrens, Alexander Waschkau, Ida Wagner Josefsson, Kristian Kidholm, Jost Steinhäuser

**Affiliations:** 1 Institute of Family Medicine, Lübeck, Germany; 2 CIMT - Centre for Innovative Medical Technology, Odense University Hospital, Odense, Denmark

**Keywords:** e health, general medicine, general practitioner, primary care, review, telemedicine

## Abstract

**Introduction:**

Telemedicine (TM) in general practice has declined following the pandemic. One reason is deemed to be the lack of clear evidence regarding the quality of care provided by TM. Therefore, the aim of this review, was to systematically present the existing evidence concerning the use of TM applications in general practice.

**Methods:**

This structured review includes randomized controlled trials (RCT) and observational studies with a control group. The literature search targeted the time period from 2011 to 2025, screened by two researchers who focused in particular on medical efficacy, economic effects, and patient experiences.

**Results:**

The searches in the PubMed and Cochrane databases yielded 488 publications (30 June 2025). After filtering out duplicates and performing multi-step screening (title, abstract and full text), 22 studies were included in the final analysis. Over 50% of these studies found positive significant effects in the clinical outcomes. Over 80% of the included studies entailed asynchronous applications and 95% of the studies observed a time period of 12 months or less. The studies focused primarily on chronic somatic diseases, including diabetes mellitus type 2, hypertension and heart failure.

**Discussion:**

Our findings demonstrate that there is evidence supporting the use of telemedicine in general practice. However, the majority of the studies focused on diseases that are not among the most common reasons for encounter and had a rather short follow-up observation period. The fact that the studies were not initiated by the specialty itself is the most likely explanation for these findings. There was no evidence that TM causes (significant) worsening of outcomes in the conditions addressed in the included studies.

## Introduction

The World Health Organization defines Telemedicine (TM) as a “cost-effective and secure use of information and communications technologies in support of health and health-related fields, including health-care services, health surveillance, health literature, and health education, knowledge and research” (1) Furthermore TM applications are defined as a synchronous (communication in real time, e.g., video consultation or phone call) or asynchronous (communication with a time delay, e.g., email) transfer of health data from patients to healthcare workers or physicians (2). This also includes TM concepts, like e.g., remote monitoring (1).

In Germany, video consultations, which were not permitted before 2017, are eligible for reimbursement since 2019 (3). Between 2020 and 2021 the percentage of statutory health insurance (SHI) physicians and psychotherapists billing for telemedical services jumped from 4.5% to 25% in comparison to 2017 (4, 5).

With the end of pandemic-related social distancing, the use of video consultations decreased in many countries. A crucial factor for this decline appears to the lack of evidence on TM based care. (3, 6)

In recent years there have been recurring studies on the quality of- and evidence for individual TM applications (7, 8) and first steps have been taken toward making the quality of TM measurable using indicators (9, 10).

With its platform TELEMED (11), the University of Southern Denmark in 2021 compiled an overview of the evidence regarding TM applications in different clinical disciplines. However, this does not include data from general practice (12).

Therefore, the aim of this review is to provide an overview of the evidence, economy and patient acceptance of TM in general medicine.

## Methods

We have performed a structured review of studies on the evidence regarding TM applications in general practice. Publications that meet the criteria of being a randomized controlled trial (RCT) or an observational study with control group were included. From these, we extracted medical efficacy, economic aspects, and patient experience. Applications in which TM communication took place between physicians and patients were included. We included both synchronous and asynchronous services. The inclusion and exclusion criteria for the study were as shown in Table 1.

**TABLE 1:** Inclusion and exclusion criteria.

Inclusion criteria	Exclusion criteria
Telemedicine study with two-way communication between patient and medical practice personnel and/or physicians	One-way communication (e.g., app with a purely informative function), communication from one physician to another physician
Randomized controlled trial (RCT) or observational study with control group	Studies which did not meet the criteria of an RCT or an observational study with control group
Study published in a peer-reviewed journal	Telemedicine services without medical personnel (e.g., coaches, etc.)
Setting: general practice	General medicine was not involved
Language: English, German	
Publication date: 12 Oct. 2011–30 June 2025	

We carried out the structured literature search in the PubMed and Cochrane databases for the period from 12 October 2011 to 30 June 2025.

The search term used was:

*((((((((telemedicine[MeSH Terms]) OR (Telemedicine)) OR (mhealth)) OR (ehealth)) OR (telehealth)) OR (“mobile health”)) OR (“home monitoring”)) AND (((((((general practice[MeSH Terms]) OR (general practitioners[MeSH Terms])) ) OR (“general practice”)) OR (“general practitioners”)) OR (“family medicine”)) OR (“general medicine”))) AND ((((((((((((RCT) OR (Controlled trial)) OR (Randomised trial)) OR (Randomized trial)) OR (Control group)) OR (Control groups)) OR (controls)) OR (control)) OR (Comparison group)) OR (Comparison groups)) OR (comparisons)) OR (comparison)).*

Filter: Study type: observational study with control group + controlled randomized trial.

Language: English + German.

All abstracts retrieved from this search were imported into the online tool “Covidence” ^1^ to sort and check the articles. The inclusion and exclusion criteria were defined in advance. Independently from each other, two researchers (a physician and a psychologist) screened all abstracts for their relevance based on the above criteria. After the title and abstract screening was complete, discussions were held to find consensus and make any final decisions about abstracts which had received differing evaluations. All abstracts deemed relevant, possibly relevant, or unclear at this stage, then underwent another round of independent evaluation through full-text screening by the researchers. After this screening was complete, a second discussion took place to reach an agreement, and all “relevant” articles were then included for data extraction, independently performed by the researchers.

The extraction was organized according to the following aspects:

1. Source: Author, year of publication, PubMed ID.
2. Setting: Country, type and number of practices/patients who participated in the study.
3. Study design: Randomized controlled trial or observational study.
4. Post-observation: Length of post-observation per patient.
5. Patient group: Number of patients in the total sample, intervention group, and control group.
6. Inclusion criteria/Exclusion criteria: The criteria described in the publication.
7. Type of technology: Description of the information and communication technology, including devices, etc.
8. Intervention: Description of the intervention including information about the aim, utilization of the service by patients, and the duration of the intervention. Measured in weeks, months, or years.
9. Clinical effects: Whether or not statistically significant differences were found between the primary and secondary outcomes. Types of outcome measures were also described.
10. Patient safety: Description of incidences or adverse reactions.
11. Patients’ experiences: Information about patients’ use and experience of the TM service.
12. Staff experiences: Information about staff experiences and amount of time spent in connection with the TM service.
13. Cost and organization: Information about financial investment, use of hospital personnel, statistically significant differences in the utilization of healthcare services between the intervention and control groups. Organizational changes in connection with the TM service were included.

The two researchers (PM and AW) met several times during the extraction phase; consensus was achieved on the data extractions during these meetings. A senior researcher (JS) was also available to resolve any remaining conflicts.

To check the methodological quality of the included studies, two researchers (PM and AW) used the Modified Downs and Black checklist for assessment of methodological quality (13) and reached a consensus during joint meetings (see Supplementary Table 2).

To make the results of this consensus more accessible, the two researchers grouped the extracted data into color-coded rankings (“traffic light system”), based on the method developed and used for the Danish TELEMED database by Kidholm et al. (10) as far as:

### Clinical effect

Green indicates a statistically significant improvement (*p* < 0.05) of the primary clinical outcome in the intervention group compared to the control group. Yellow means that no statistically significant difference was seen in the primary outcome, and red indicates a statistically significant worsening of the primary clinical outcome.

If a study includes several primary outcomes, green means that one statistically significant improvement was found for at least one outcome and none for the other outcomes. If short-term and long-term clinical outcomes are described in the same publication, the information concerning the long-term outcomes was used as the basis for assessment.

### Patient experiences

Green means that more than 50% of the patients were satisfied with the service or that more than 50% of the patients in the intervention group used the TM service. Yellow indicates mixed results in terms of the patients’ perceptions, e.g., more than 50% of the patients in the intervention group were satisfied but less than 50% of the patients used the service. Red means that less than 50% of the patients expressed satisfaction with the service or that less than 50% of the patients in the intervention group used the TM service.

### Economic effects

Green indicates a statistically significant decrease (*p* < 0.05) in healthcare utilization by patients in the intervention group compared to the control group. Yellow means no statistically significant change in the utilization of healthcare, and red shows a statistically significant increase in healthcare utilization by the patients. If a study has multiple measures for healthcare utilization, the color green indicates a statistically significant decrease in at least one measure and no effects on other measures. The color yellow means a statistically significant decrease or increase in healthcare utilization, and red means that at least one measure for healthcare utilization in the intervention group had a statistically significantly increase and other measures showed no significant changes.

The color gray was used when the publication did not contain any information about the effects.

In addition to the color-coded rankings, information was also gathered on the challenges connected with implementing the TM services described in the separate publications. This analysis was carried out separately by two researchers with subsequent meetings to achieve consensus. These challenges were classified as follows:

1. The TM service requires cooperation between organizations or healthcare sectors (e.g., between general practitioner (GP) and commune) (A).
2. The TM service requires acquisition of new IT equipment (e.g., devices to measure blood pressure) which patients cannot be expected to already own themselves (B).
3. The TM service requires the development of new IT solutions (e.g., web pages or apps) (C).
4. The TM service requires the training of healthcare workers (e.g., training to use IT devices that are not otherwise used by the workers) (D).
5. The TM services require extra amounts of time to be spent by healthcare workers (e.g., time for extra video consultations) (E).
6. The TM service requires integration into the local electronic patient data systems (e.g., to ensure scheduled GPs appointments are included in a new patient app) (F) (10).

Further information was extracted in addition to this, namely, which of the included studies contained asynchronous or synchronous TM services, if the lead author or principal investigator came from the field of general medicine, as well as how many and which MAST domains (model for assessment of telemedicine applications) were included.

## Results

**FIGURE 1:**
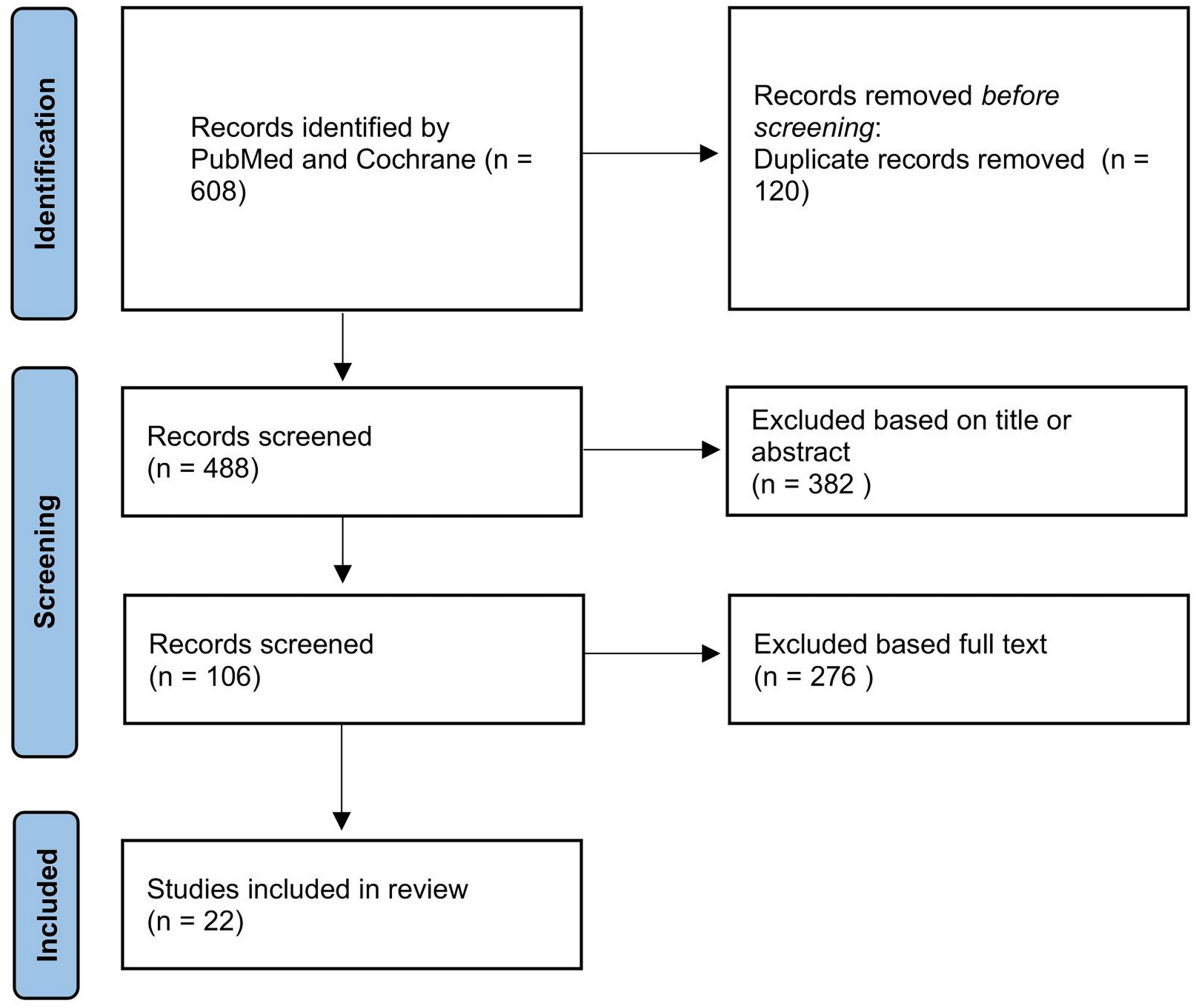
The flowchart illustrates the process of identifying, screening and including the articles.

After subtracting out the duplicates, the structured search yielded 488 publications. Following title and abstract screening (most recent screening on 30 June 2025), 106 publications were included in the full-text screening. In this screening step, 22 publications were identified for inclusion in the data extraction step (see Figure 1).

Here, 18 studies involved an asynchronous TM service, two a synchronous TM service, and two offered a mixed synchronous/asynchronous TM service (Table 2). All of the telemedicine applications examined utilized multiple technologies (e.g., Home monitoring and website), with the exception of one included study, which involved only a phone call (see Supplementary Table 1). The control groups received standard care.

A quality analysis of the selected studies using the modified checklist of Black and Down rated 19 of the 22 studies as good quality, with scores of 20–24 points (good = 20–25 points), and three studies as fair quality, with scores of 19 points (fair = 15–19 points).

In 60% of publications, no author from general practice was listed first or last in the list of authors.

The following lists the number of times a clinical topic was investigated: diabetes mellitus type II: 6; arterial hypertension: 4; multiple responses: 2 (COPD/heart failure/diabetes mellitus type II); heart failure: 2; asthma (pediatric): 1; chronic full-body pain: 1; COPD: 1; COVID-19: 1; dermatology: 1; metabolic syndrome: 1; orthopedic problems: 1; psychological problems: 1.

In 14 studies a positive effect (63.6%) was seen in the clinical outcomes defined as primary outcomes when comparing the intervention groups (use of TM procedures) with the control groups, and five studies found no effect (22.7%). A decline in the clinical situation was not seen in any of the studies. Data regarding clinical effect were missing in three studies (13.6%).

In regard to the patient experience, three studies (13.6%) were able to describe a significant positive finding, whereas all of the other studies had no information on this aspect.

In regard to the economic aspects, three studies showed a positive effect (13,6%), three studies found no effect (13.6%), and four studies a negative effect (18%). A total of 12 studies (54.5%) contained no information.

The primary outcomes in each of these studies are grouped as described in Table 2.

**TABLE 2:** Information about author, title, clinical topic, type of TM (asynchrone/synchrone), MAST-criteria, follow-up, outcomes and background of authors of included studies.

No.	Author	Title	Clinical topic	Async hrone (A)/synchrone (S)	MAST-criteria taken into account	Follow-up	Outcomes	Background of first and last author
1	Bardsley et al.	Impact of telehealth on general practice contacts: findings from the whole systems demonstratorcluster randomized trial	COPD, heart-failure, diabetes	A	1,3,6	12 months	Frequency of visits to GP	No GP
2	Basudev et al.	A prospective randomized controlled study of a virtual clinic integrating primary and specialist care for patients with type 2 diabetes mellitus	Type-2-diabetes	A/S (virtually clinic/patient consultation)	1,3,6	12 months	- HbAlc - Blood pressure	No GP
3	Bujnowska-Fedak et al.	The impact of telehome care on health status and quality of life among patients with diabetes in a primary care setting in Poland	Type-2-diabetes	A	1,3,4,6	6 months	- HbAlc - Hypoglycemic events - hyperglycemic events - Higher overall scores on QoL measures	Department of Family Medicine, Poland
4	Cartwright et **al.**	Effect of telehealth on quality of life and psychological outcomes over 12 months (whole systems demonstrator telehealth questionnaire study): nested study of patient reported outcomes in a pragmatic, cluster randomized controlled trial	COPD, heart-failure, diabetes (QoL)	A	1, 2, 3, 4, 6	12 months	-QoL - Anxiety - Depressive symptoms	No GP
5	Dendale et al.	Effect of a telemonitoring-facilitated collaboration between general practitioner and heart failure clinic on mortality and rehospitalization rates in severe heart failure: the TEMA-HF1 (telemonitoring in the management of heart failure) study	Chronic-heart-failure	A	1,3,6	6 months	- Mortality - Heart failure-related hospitalization - Reduced days lost to death, dialysis, or hospitalization for heart or renal failure - LVEF and NT-proBNP	No GP
6	Farmer et al.	Self-management support using a digital health system compared with usual care for chronic obstructive pulmonary disease: randomized controlled trial	COPD	A	1,3,6	12 months	- Change in St George’s Respiratory Questionnaire for COPD (SGRQ-C)	First author: Institute of Family Medicine last author: no GP
7	Gidding et al.	PsyScan e-tool to support diagnosis and management of psychological problems in general practice: a randomized controlled trial	Psychological problems	A	1,3,6	12 months	- Chances of achieving symptom reduction (SCL-90-R) of ≥50% after 1 year	GP
8	Hoffmann-Petersen et al.	Short-term telemedical home blood pressure monitoring does not improve blood pressure in uncomplicated hypertensive patients	Hypertension/blood pressure	A	1,3,6	3 months	Blood pressure	Only second author: Institute of Family Medicine All others: nephrologists
9	Krum et al.	Telephone support to rural and remote patients with heart failure: the chronic heart failure assessment by telephone (CHAT) study	Heart failure	*A/S*	1,3,6	12 months	Packer clinical composite score	No GP
10	McManus et al.	Home and online management and evaluation of blood pressure (HOME BP) using a digital intervention in poorly controlled hypertension: randomized controlled trial	Hypertension/blood pressure	A	1, 2, 3, 5,6	12 months	Blood pressure	First author: Institute of Family Medicine last author: no GP
11	McManus et al.	Efficacy of self-monitored blood pressure, with or without telemonitoring, for titration of antihypertensive medication (TASMINH4): an unmasked randomized controlled trial	Hypertension/blood pressure	A	1, 2, 3, 6	12 months	Blood pressure	GP
12	Nicolucci et al.	A randomized trial on home telemonitoring for the management of metabolic and cardiovascular risk in patients with type 2 diabetes	Type-2-diabetes	A	1, 2, 3, 6	12 months	HbAlc [body weight, blood pressure, lipids, health related QoL (SF-36)]	No GP
13	Odnoletkoval et al.	People with type 2 diabetes, affiliated to the Belgian health insurance fund “Partena,” were selected based on their glucose-lowering medication consumption, invited into the study by Partena	Type-2-diabetes	A	1, 3, 5, 6	18 months	HbAlc (6 months) [BMI, weight, total cholesterol, LDL, HDL, triglyceride, systolic BP, diastolic BP, EQ-5D (health utilities) 18 months]	No GP first and last among the authors
14	Petrella et al.	Community dwelling adults were recruited via print and radio advertisements, word of mouth, community presentations and physician referral	Metabolic syndrome	A	1,3,6	52 weeks	Blood pressure (diastolic blood pressure, waist circumference, HbAlC, LDL, cholestrol, triglycerides, HDL, fasting plasma glucose, HOMA-IR, CRPhs)	Institute of Family Medicine
15	Shaoxia et al.	A randomized asthma controlled trial of a mobile application-assisted nurse-led model used to improve treatment outcomes in children with asthma	Asthma (children)	A	1,3,5,6	12 months	Asthma exazerbations (treatment adherence, C-ACT score, required oral steroid treatment, days of antibiotic use, frequency of respiratory tract infections, days of school absence)	First author: Institute of Family Medicine last author: no GP
16	Steventon et al.	Effect of telehealth on glycemic control: analysis of patients with type 2 diabetes in the whole systems demonstrator cluster randomized trial	Diabetes	A	1,3,6	12 months	HbAlc	No GP
17	Leupold et al.	Digital redesign of hypertension management with practice and patient apps or blood pressure control (PIA study): a cluster-randomized controlled trial in general practices	Hypertension/blood pressure	A	1,3,6	12 month	Blood pressure	Institute of Family Medicine
18	Guo et al.	Effectiveness of mHealth management with an implantable glucose sensor and a mobile application among Chinese adults with type 2 diabetes	Diabetes	A	1,3,6	4 weeks	-BMI - FBG (fasting blood glucose) - 2hPG (postprandial two-hour blood glucose) - HbAlc -QoL - Self management	No GP
19	Gyllensten et al.	Physical activity with person-centered guidance supported by a digital platform or with telephone follow-up for persons with chronic widespread pain: health economic considerations along a randomized controlled trial	Chronic widespread pain	A	1,3,5,6	12 months	Cost comparison	No GP
20	Foni et al.	Guideline-based telemedicine assessment of orthopedic low-risk conditions by general practitioners is not inferior to that of face-to-face consultations with specialists in the Emergency department: a randomized trial	Orthopedic low risk conditions	S	1,3,4,6	12 months	Comparison: syndromic diagnosis, physical examination profile regarding inspection palpation data, red flag identification, beyond-order tests, suggested medications, proposed destinations	No GP
21	Sauvage et al.	6-month outcomes after a GP phone call during the first French COVID-19 lockdown (COVIQuest): a cluster randomised trial using medico- administrative databases	- Chronic diseases/COVID 19	S	1,3,5,6	6 months	Hospital admissions	GP
22	Lopez-Villegas et al.	Cost-utility analysis of teledermatology units in primary care centers versus face-to-face dermatology consultations in the hospital	- Dermatology	A	1,4,5,6	6 months	- Total costs	No GP

Of the 22 included studies, 15 studies had a follow-up of 12 months, 4 studies had one of 6 months, and there was one study each for 1 month, 3 months and 18 months.

Overall, the studies applied very different assessment scores, e.g., for quality of life (QoL).

## Discussion

It was possible to identify TM studies from general medicine whose results indicate that an improvement in clinical outcomes occurred. Economic information was less frequently reported in comparison. As yet patients’ perspectives have hardly been considered.

Most of the investigated TM applications were asynchronous, fitting to the finding that asynchronous TM is perceived as particularly helpful (14).

The MAST domains provide a structured model for assessing TM applications and were developed at the behest of the European Commission (15). Present in all of the studies were domains 1 (Health problem and characteristics of the application), 3 (Clinical effectiveness), and 6 (Organizational aspects) – already defined in advance by the inclusion criteria. In regard to the multidisciplinary assessments, it was only very seldom that any were carried out [e.g., point 4 (Patient perspective)] or they were absent in all of the included studies [point 7 (Socio-cultural, ethical and legal aspects)]. Previous studies also confirm this limitation (6).

The topics addressed with TM are not the ones most frequently seen in primary care (16). Instead, they are mostly in areas where TM studies already exist. A clear majority of these studies focus on cardiology, followed by pneumology and endocrinology (10). In contrast, a large study on reasons for primary care visits shows that endocrinological and cardiovascular conditions, combined, account for only 3% (13). This gap might be explained by the finding that general practitioners were mostly not the primary responsible person in the research project.

The follow-up periods are rather brief, particularly when looking at chronic diseases. Also, when looking at secondary outcomes, only one study with an observation period of 18 months was identified. Thus, none of the included studies supply longitudinal results in the way they would be adequate for general practitioners, since long-term, continued and comprehensive care is characteristic of this area of medicine (17).

In the future it would be desirable to more strongly involve general practitioners in the study design in order to represent aspects with more relevance to primary care, e.g., polypharmacy.

Uniform outcomes measures, e.g., on QoL, number of sick days and length of incapacity to work, mortality, medication and treatment costs, referrals, hospital admissions, avoidance of travel (time and expense savings), etc., in the form of longer-term parameters would be useful.

Furthermore, it would be desirable for future TM studies to focus more on the patient perspective: Only three of the included studies examined the patients’ perspectives. When implementing TM applications, it is also conducive to consider what patients need from these products (18). Earlier studies show that, in particular, patients in rural areas display a lower affinity compared to younger city dwellers (5).

In this study we have analyzed publications written in English or German only. It is possible that a few studies were missed for not fulfilling this criterion but which otherwise may have met the remaining inclusion criteria. However, one strength of the included studies is that they come from 14 different countries around the world. To have been included in our study, communication had to have taken place between the physician and patient (“doctor-to-patient,” d2p). Applications that entailed communication between physicians were excluded (“doctor-to-doctor,” d2d). Studies show that there could be advantages for general medicine precisely in asynchronous d2p applications (11). The “traffic light” system, which we used based on previously published data on telemedicine applications in other medical specialties (11), is quite coarse, allowing small statistical differences to influence the results. It serves to provide an overview.

Given the fact that about 80% of all reasons for encounter are solved within general practice, 22 studies seem a rather limited amount of evidence. However, there was no notice that TM causes (significant) worsening of outcomes. Future TM projects in general practice should be developed by the specialty itself.

## Footnotes

1
https://www.covidence.org/
