# Supplementary material for: Telemedicine applications in general medicine - a structured review of the evidence
Source: Front Med (Lausanne). 2026 Apr 24;13:1771532. doi: 10.3389/fmed.2026.1771532 (PMC13152840; doi:10.3389/fmed.2026.1771532)
Supplement: Supplementary file 1 [file Supplementary_file_1.pdf]

Suppl. 1: Traffic Light System

Nr.	Title	Country	Diagnosis	Intervention	Technology	Clinical Effect	Patient Experience	Economic Effect	Implementation
1	Impact of telehealth on general practice contacts: findings from the whole systems demonstratorcluster randomised trial	GB	- COPD<br>- heart failure<br>- diabetes	Flexibility for local teams to develop their own telehealth services choices of telehealth devices and monitoring systems varied between the three trial sites and there was no attempt to standardise across sites	- Home monitoring<br>- SMS / Mail	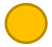	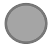	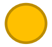	B, C, D, E
2	A prospective randomized controlled study of a virtual clinic integrating primary and specialist care for patients with	GB	- diabetes typ II	Virtual diabetic clinic, with education and monitoring of vital data	- Phone consultation - Home monitoring<br>- SMS / E-Mail	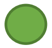	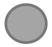	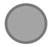	B,C,D,E

Suppl. 1: Traffic Light System

	Type 2 diabetes mellitus								
3	The Impact of Telehome Care on Health Status and Quality of Life Among Patients with Diabetes in a Primary Care Setting in Poland	Poland	- diabetes typ II	- telehome monitoring system to collect measurement of blood glucose<br>- GPs could analyze data and were provided with decision support flagginng of critical conditions	- Home monitorin g<br>- Website<br>- Phone consultati on	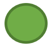	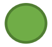	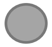	B, C, D, E
4	Effect of telehealth on quality of life and psychologicaloutco mes over 12 months (Whole Systems Demonstrator telehealth questionnaire study): nested study of patient reported outcomes in a pragmatic, cluster randomised controlled trial	GB	- COPD<br>- diabetes<br>- heart failure	Synchronous transfer of Data with some real time processing of patient data. Care providers can recognise important changes in essential measurements, but delays can occur if the systems are only active during office hours.	- Home monitorin g<br>- Website<br>- Phone consultati on	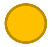	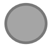	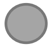	B, C, D, E

Suppl. 1: Traffic Light System

5	Effect of a telemonitoring-facilitated collaboration between general practitioner and heart failure clinic on mortality and rehospitalization rates in severe heart failure: the TEMA-HF 1 (TElemonitoring in the MAnagement of Heart Failure) study	Belgium	- cronic heart failure	- Short education<br>- measurement of body weight, blood pressure, and heart rate daily, at fixed hour in the morning<br>- missing of transmission on two days resulted in visit by GP (if necessary)<br>- contact by nurse 1-3 days after such an incident	- Phone consultation<br>- Home monitoring -<br>SMS/Mail<br>- Website	🟢	⚪	🟡	A,B,C,D,E
6	Self-Management Support Using a Digital Health System Compared With Usual Care for Chronic Obstructive Pulmonary Disease: Randomized Controlled Trial	GB	- COPD	Patients with COPD recieved tablet, EDGE-platform and bluetooth-pulsoximeter to help patients identify exacerbations and to monitor their condition. Also they recieved self-	- Phone consultation - Home monitoring - Website	🟡	⚪	⚪	B,C,D,E

				management-support					
7	PsyScan e-tool to support diagnosis and managementof psychological problems in general practice: a randomised controlled trial	Netherlands	psychological problems	E-tool that patients can use to get therapeutic advice between consultations with their GP	- Home monitoring - Website				B,C,D,E
8	Short-term telemedical home blood pressure monitoring does not improve blood pressure in uncomplicated hypertensivepatients	Denmark	- hypertension	Telemonitoring of home blood pressure measurements (TBPM). Antihypertensive treatment was based on TBPM with transmission of the measurements and subsequent communication by telephone or e-mail	- Phone consultation - Home monitoring - SMS/Mail - Website				B, C, D, E
9	Telephone Support to Rural and Remote Patients with Heart Failure:	Australia	- heart failure	- ongoing support by touchtone telephone using	- Phone consultation				B, C, D, E

	The Chronic Heart failure Assessment by Telephone (CHAT) study			the TeleWatch™ system, recieved<br>- an action plan<br>- information resources, e.g newsletters<br>- individualised diary	- Home monitorin g - Website				
10	Home and Online Management and Evaluation of Blood Pressure (HOME BP) using a digital intervention in poorly controlled hypertensio : randomised controlled trial	GB	- hypertension	- The intervention consisted of an integrated patient and healthcare practitioner online digital intervention, blood pressure self-monitoring, healthcare practitioner directed and supervised titration of antihypertensive drugs, and user selected lifestyle modifications.	- Phone consultati on<br>- Home monitorin g - SMS/Mail - Website	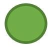	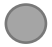	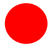	B, C, D, E
11	Efficacy of self-monitored blood pressure, with or	GB	- hypertension	Participants randomly assigned to self-	- Phone consultati on	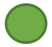	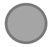	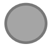	B, C, D, E

	without telemonitoring, for titration of antihypertensive medication (TASMINH4): an unmasked randomised controlled trial			monitoring were taught to use a validated automated electronic sphygmomanometer checking with a colour chart. Participants in the telemonitoring group were trained to send readings via a simple free SMS text-based telemonitoring service with web-based data entry back-up. The telemonitoring system incorporated an algorithm that alerted participants to contact their surgery in the light of very high or very low readings,	- Home monitoring - SMS/Mail - Website				

				reminded them if insufficient readings were transmitted, prompted them to make contact with their practice if their average blood pressure was above target, and presented readings to attending clinicians via a web interface					
12	A Randomized Trial on Home Telemonitoring for the Management of Metabolic and Cardiovascular Risk in Patients with Type 2 Diabetes	Italy	- diabetes typ II	- Home telehealth system<br>- monthly contact by nurses	- Phone consultation<br>- Home monitoring - SMS/Mail - Website	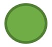	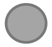	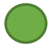	B, C, D, E
13	Optimizing diabetes control in people with Type 2 diabetes through nurse-led telecoaching	Belgium	- diabetes typ II	- patients recieved COACH programme (identifies the ‘treatment gaps’ in the management of	- Phone consultation<br>- Home monitoring	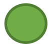	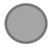	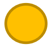	B, C, D, E

				each diabetes risk factor, i.e. failure to achieve the guideline-recommended goals, and helps the patient to identify strategies to close the treatment gap, including lifestyle adjustments and adherence to recommended medication therapy)	-SMS/Mail<br>- Website				
14	Mobile health, exercise and metabolic risk: a randomized controlled trial	Canada	- at least two metabolic syndrome risk factors	interventiongrou p recieves exercise prescription complied with current global physical activity guidelines and homemonitoring equipment to monitor health data. When measurements were outside	- App<br>- Home monitorin g				B, C, D, E

				of pre-determined safety limits, an automated alert was sent to the study physician's smartphone for followup					
15	A randomized controlled trial of a mobile application-assisted nurse-led model used to improve treatment outcomes in children with asthma	China	- asthma	Children in the two groups visited their paediatricians at four hospitals once a month for a total of 12 months. Two weeks after visit nurse contacted parents for review of data und reminding oi usage of app.	- Phone consultati on<br>- Home monitorin g<br>- App	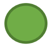	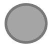	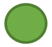	B, C, D, E
16	Effect of telehealth on glycaemic control: analysis of patients with type 2 diabetes in the Whole Systems Demonstrator	GB	-diabetes	Telehealth equipment reminded participants to take physiological For example, participants with diabetes and	- Home monitorin g<br>- Website	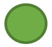	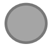	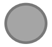	B, C, D, E

	cluster randomised trial			well-controlled blood glucose were typically asked to take readings less frequently than participants whose blood glucose was poorly controlled.					
17	Digital redesign of hypertension management with practice and patient apps for blood pressure control (PIA study): a cluster-randomised controlled trial in general practices	GER	hypertension	PIA is a complex intervention comprising two elements: the PIA-ICT (PIA app and PIA-PrMC) and the PIA education (eLearning/on-site training for practice teams and patients)	- App<br>- Home monitorin g	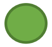	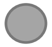	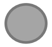	B, C, D, E
18	Effectiveness of mHealth managementwith an implantable glucose sensorand a mobile application amongChinese adults with type 2 diabetes	China	- diabetes	Intervention group received mHealth management based on the mHealth management model that consisted of the	- App<br>- Home monitorin g	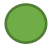	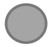	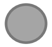	B, C, D, E

				network platform, an implantable glucose sensor, a mobile app and GP support.					
19	Physical activity with person-centered guidance supported by a digital platform or with telephone follow-up for persons with chronic widespread pain: Health economic considerations along a randomized controlled trial	Sweden	- chronic widespread pin	Investigation of the resource use and costs associated with co-creation of a person-centered health-enhancing physical activity plan for CWP-patients followed by support through adidgital platform, compared to teleohone follow-up	- App, - Phone consultation<br>- Home monitoring				B, C, D, E
20	Guideline-Based Telemedicine Assessment of Orthopedic Low-Risk Conditions by General Practitioners is Not	Brazil	- orthopedic low risk conditions	Patients were randomly assigned to receive a medical consultation via TM with a GP, followed by a	- website<br>-App				B, C, D, E

	Inferior to that of Face-to-Face Consultations with Specialists in the Emergency Department: A Randomized Trial			subsequent in-person assessment from an orthopedist (TM group) or a direct in-person assessment from an orthopedist (IPgroup)					
21	Six- month outcomes after a GP phone call during the first French COVID- 19 lockdown (COVIQuest): a cluster randomised trial using medico-administrative databases	France	- chronic diseases / Covid19	The COVIQuest project was thus developed in order to assess the effect of a phone call by a GP or a medical trainee during the first lockdown on the care pathway of chronic patients	- Phone consultation	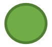	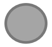	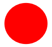	C, D, E
22	Cost-Utility Analysis of Teledermatology Units in Primary Care Centers Versus Face-to-Face DermatologyConsultations in the Hospital	Spain	- dermatology	The main objective of this study was to perform a cost-utility analysis to determine whether TD units in PC-centers offer an alternative in	- Home monitoring<br>- Website	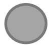	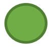	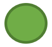	B, C, D, E

				terms of cost and quality of life to conventional F-F/D performed in the hospital.					

Colour	Category
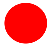	Negative Effect
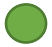	Positive Effect
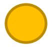	No Effect
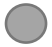	No information

Technology:

App , Video consultation, Phone consultation , Home monitoring , SMS/Mail , Website
