# Supplementary material for: Telemedicine applications in general medicine - a structured review of the evidence
Source: Front Med (Lausanne). 2026 Apr 24;13:1771532. doi: 10.3389/fmed.2026.1771532 (PMC13152840; doi:10.3389/fmed.2026.1771532)
Supplement: Supplementary file 2 [file Supplementary_file_2.pdf]

Modified* Downs and Black checklist for assessment of methodological quality.

Studies were classified as excellent (26–28), good (20–25), fair (15–19), and poor (≤ 14) quality [1,2].

The modified Downs and Black checklist (Item - Modified Downs and Black checklist for assessment of methodological quality. - figshare - Figshare)

Item	Criteria	Answers	1	2	3	4	5	6	7	8	9	10	11	12	13	14	15	16	17	18	19	20	21	22
**Reporting**			**Bardsley (2013)**	**Basudev (2015)**	**Buinowska-Fedak (2011)**	**Cartwright (2013)**	**Dendale (2011)**	**Farmer (2017)**	**Gidding (2018)**	**Hoffmann-Petersen (2016)**	**Krum (2012)**	**McManus (2020)**	**McManus (2018)**	**Nicolucci (2015)**	**Odnoletkova (2016)**	**Petrella (2014)**	**Shaoxia (2018)**	**Stevenson (2014)**	**Leupold (2022)**	**Guo (2021)**	**Gyllensten (2024)**	**Foni (2024)**	**Sauvage (2024)**	**Lopez-Villegas (2024)**
1	*Is the hypothesis/aim/objective of the study clearly described?*	Yes = 1 No = 0	1	1	1	1	1	1	1	1	1	1	1	1	1	1	1	1	1	1	1	1	1	1

2	*Are the main outcomes to be measured clearly described in the Introduction or Methods section?* If the main outcomes are first mentioned in the Results section, the question should be answered no.	Yes = 1 No = 0	1	1	1	1	1	1	1	1	1	1	1	1	1	1	1	1	1	1	1	1	1	1
3	*Are the characteristics of the patients*	Yes = 1 No = 0	1	1	1	1	1	1	1	1	1	1	1	1	1	1	1	1	1	1	1	1	1	1

	included in the study clearly described? In cohort studies and trials, inclusion and/or exclusion criteria should be given. In case-control studies, a case-definition and the source for controls should be given.																							
4	*Are the interven*	Yes = 1	1	1	1	1	1	1	1	1	1	1	1	1	1	1	1	1	1	1	1	1	1	1

	tions of interest clearly described? Treatments and placebo (where relevant) that are to be compared should be clearly described.	No = 0																						
5	*Are the distributions of principal confounders in each group of subjects to be compared clearly described?*	Yes = 2 Partially = 1 No = 0	1	2	1	1	2	1	1	0	0	2	0	0	1	0	0	0	0	1	0	0	0	1

	A list of principal confounders is provided.																							
6	*Are the main findings of the study clearly described?* Simple outcome data (including denominators and numerators) should be reported for all major findings so that the reader can	Yes = 1 No = 0	1	1	1	1	1	1	1	1	1	1	1	1	1	1	1	1	1	1	1	1	1	1

	check the major analyses and conclusions. (This question does not cover statistical tests which are considered below).																							
7	*Does the study provide estimates of the random variability in the data for the main outcomes?* In non-normally	Yes = 1 No = 0	1	1	1	1	1	1	1	1	1	1	1	1	1	1	1	1	1	1	0	1	1	1

	distributed data the interquartile range of results should be reported. In normally distributed data the standard error, standard deviation or confidence intervals should be reported. If the distribution of the data is not describe																							

	d, it must be assumed that the estimates used were appropriate and the question should be answered yes.																							
8	*Have all important adverse events that may be a consequence of the intervention been reported?* This should	Yes = 1 No = 0	0	0	0	1	0	1	0	0	0	1	1	1	0	1	0	0	0	0	0	0	0	0

	be answered yes if the study demonstrates that there was a comprehensive attempt to measure adverse events. (A list of possible adverse events is provided).																							
9	*Have the characteristics of patients lost to follow-up been described?*	Yes = 1 No = 0	1	1	1	1	1	1	1	0	0	1	1	1	1	1	1	1	1	0	1	1	1	1

	This should be answered yes where there were no losses to follow-up or where losses to follow-up were so small that findings would be unaffected by their inclusion. This should be answered ‘no’ where a study does not report the																							

	number of patients lost to follow-up.																							
10	*Have actual probability values been reported (e.g. 0.035 rather than <0.05) for the main outcomes except where the probability value is less than 0.001?*	Yes = 1 No = 0	1	1	1	1	1	1	1	1	1	1	1	1	1	1	1	1	1	1	0	1	1	1
**External validity**																								
11	*Were the subjects asked to*	Yes = 1 No = 0	1	1	1	1	1	1	1	1	1	1	1	1	1	1	1	1	1	1	1	1	1	1

	participate in the study representative of the entire population from which they were recruited? The study must identify the source population for patients and describe how the patients were selected. Patients would be	Unable to determine = 0																						

	representative if they comprised the entire source population, an unselected sample of consecutive patients, or a random sample. Random sampling is only feasible where a list of all members of the relevant population exists. Where a study does not																							

	report the proportion of the source population from which the patients are derived, the question should be answered as unable to determine.																							
12	*Were those subjects who were prepared to participate representative of*	Yes = 1 No = 0 Unable to determi	1	1	1	1	1	1	1	1	1	1	1	1	1	1	1	1	1	1	1	1	1	1

	the entire population from which they were recruited? The proportion of those asked who agreed should be stated. Validation that the sample was representative would include demonstrating that the distribution of the	ne = 0																						

	main confounding factors was the same in the study sample and the source population.																							
13	*Were the staff, places, and facilities where the patients were treated, representative of the treatment the majority of patients receive?*	Yes = 1 No = 0 Unable to determine = 0	1	1	1	1	1	1	1	1	1	1	1	1	1	1	1	1	1	1	1	1	1	1

	For the question to be answered yes the study should demonstrate that the intervention was representative of that in use in the source population. The question should be answered no if, for example, the intervention was undertaken in a specialis																							

	t centre unrepresentative of the hospitals most of the source population would attend.																							
**Internal validity - bias**																								
14	*Was an attempt made to blind study subjects to the intervention they have received?* For studies where the patients would have no	Yes = 1 No = 0 Unable to determine = 0	0	0	0	0	0	0	0	0	0	0	0	0	0	0	0	0	0	0	1	0	0	0

	way of knowing which intervention they received, this should be answered yes.																							
15	*Was an attempt made to blind those measuring the main outcomes of the intervention?*	Yes = 1 No = 0 Unable to determine = 0	0	1	0	0	0	0	0	0	1	0	0	0	1	0	0	0	0	0	0	1	0	0
16	*If any of the results of the study were based on “data dredgin*	Yes = 1 No = 0 Unable to det	1	1	1	1	1	1	1	1	1	1	1	1	1	1	1	1	1	1	1	1	1	1

	g”, was this made clear? Any analyses that had not been planned at the outset of the study should be clearly indicated. If no retrospective unplanned subgroup analyses were reported, then answer yes.	ermine = 0																						
17	*In trials and cohort*	Yes = 1	1	1	1	1	1	1	1	1	1	1	1	1	1	1	1	1	1	1	1	1	1	1

	studies, do the analyses adjust for different lengths of follow-up of patients, or in case-control studies, is the time period between the intervention and outcome the same for cases and controls? Where follow-up was the same for	No = 0 Unable to determine = 0																						

	all study patients the answer should be yes. If different lengths of follow-up were adjusted for by, for example, survival analysis the answer should be yes. Studies where differences in follow-up are ignored should be answered no.																							

18	*Were the statistical tests used to assess the main outcomes appropriate?* The statistical techniques used must be appropriate to the data. For example nonparametric methods should be used for small sample sizes. Where little	Yes = 1 No = 0 Unable to determine = 0	1	1	1	1	1	1	1	1	1	1	1	1	1	1	1	1	1	1	1	1	1	1

	statistical analysis has been undertaken but where there is no evidence of bias, the question should be answered yes. If the distribution of the data (normal or not) is not described it must be assumed that the estimates used were																							

	appropriate and the question should be answered yes.																							
19	*Was compliance with the intervention/s reliable?* Where there was noncompliance with the allocated treatment or where there was contamination of one group, the question	Yes = 1 No = 0 Unable to determine = 0	1	0	1	1	1	1	1	0	1	1	0	1	1	1	1	1	1	1	1	0	0	1

	should be answered no. For studies where the effect of any misclassification was likely to bias any association to the null, the question should be answered yes.																							
20	*Were the main outcome measures used accurate (valid and*	Yes = 1 No = 0 Unable to deter	1	1	1	1	1	1	1	1	1	1	1	1	1	1	1	1	1	1	1	1	1	1

	reliable)? For studies where the outcome measures are clearly described, the question should be answered yes. For studies which refer to other work or that demonstrates the outcome measures are accurate, the	mine = 0																						

	question should be answered as yes.																							
**Internal validity – confounding (selection bias)**																								
21	*Were the patients in different intervention groups (trials and cohort studies) or were the cases and controls (case-control studies) recruited from the same population?*	Yes = 1 No = 0 Unable to determine = 0	1	1	1	1	1	1	1	1	1	1	1	1	1	1	1	1	1	1	1	1	1	1

	For example, patients for all comparison groups should be selected from the same hospital. The question should be answered unable to determine for cohort and case-control studies where there is no information concerni																							

	ng the source of patients included in the study.																							
22	*Were study subjects in different intervention groups (trials and cohort studies) or were the cases and controls (case-control studies) recruited over the same period of time?* For a	Yes = 1<br>No = 0<br>Unable to determine = 0	1	1	1	1	1	1	1	1	1	1	1	1	1	1	1	1	1	1	1	1	1	1

	study which does not specify the time period over which patients were recruited, the question should be answered as unable to determine.																							
23	*Were study subjects randomized to intervention groups?* Studies which state that subjects	Yes = 1 No = 0 Unable to determine = 0	1	1	1	1	1	1	1	1	1	1	1	1	1	1	1	1	1	1	1	1	1	1

	were randomized should be answered yes except where method of randomization would not ensure random allocation. For example alternate allocation would score no because it is predictable.																							
24	*Was the randomized intervention*	Yes = 1 No = 0	0	0	0	0	0	0	0	0	0	0	0	0	0	0	0	0	0	0	0	1	0	0

	assignment concealed from both patients and health care staff until recruitment was complete and irrevocable? All nonrandomized studies should be answered no. If assignment was concealed from patients but not from staff, it should	Unable to determine = 0																						

	be answered no.																							
25	*Was there adequate adjustment for confounding in the analyses from which the main findings were drawn?* This question should be answered no for trials if: the main conclusions of the study were	Yes = 1 No = 0 Unable to determine = 0	0	0	0	0	0	0	0	0	0	0	0	0	0	0	0	0	0	0	0	0	0	0

	based on analyses of treatment rather than intention to treat; the distribution of known confounders in the different treatment groups was not described; or the distribution of known confounders differed between the treatme																							

	nt groups but was not taken into account in the analyses. In non-randomized studies if the effect of the main confounders was not investigated or confounding was demonstrated but no adjustment was made in the final analyses the question																							

	should be answered as no.																							
26	*Were losses of patients to follow-up taken into account?* If the numbers of patients lost to follow-up are not reported, the question should be answered as unable to determine. If the proporti	Yes = 1 No = 0 Unable to determine = 0	1	1	1	1	1	1	1	1	1	1	1	1	1	1	1	1	1	1	1	1	1	1

	on lost to follow-up was too small to affect the main findings, the question should be answered yes.																							
**Power**																								
27*	*Was a power analysis performed?*	Yes = 1 No = 0 Unable to determine = 0	0	0	0	1	0	1	1	1	0	1	1	1	0	1	1	0	1	1	0	1	0	1
**Sum**			**21**	**22**	**21**	**23**	**22**	**23**	**22**	**19**	**20**	**24**	**21**	**22**	**22**	**22**	**21**	**20**	**21**	**21**	**19**	**22**	**19**	**22**

**m<br>e**																								

*Modified from the original form published by Downs and Black.
